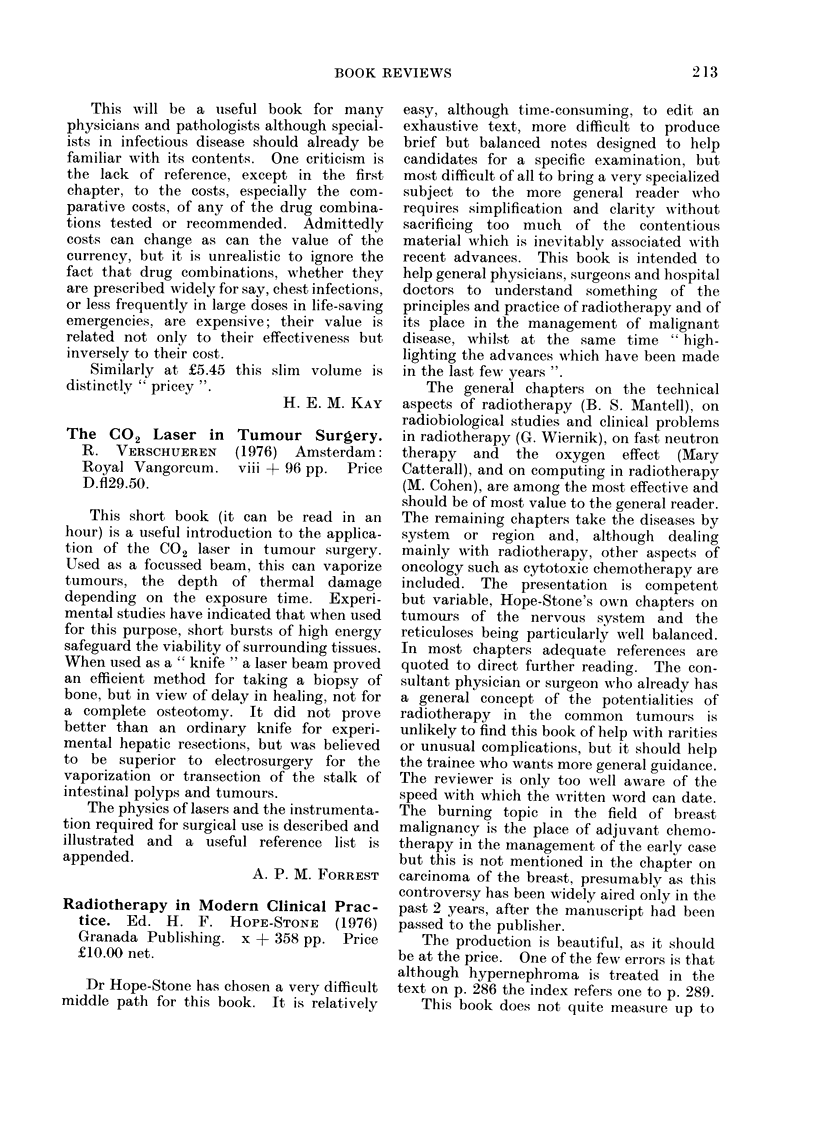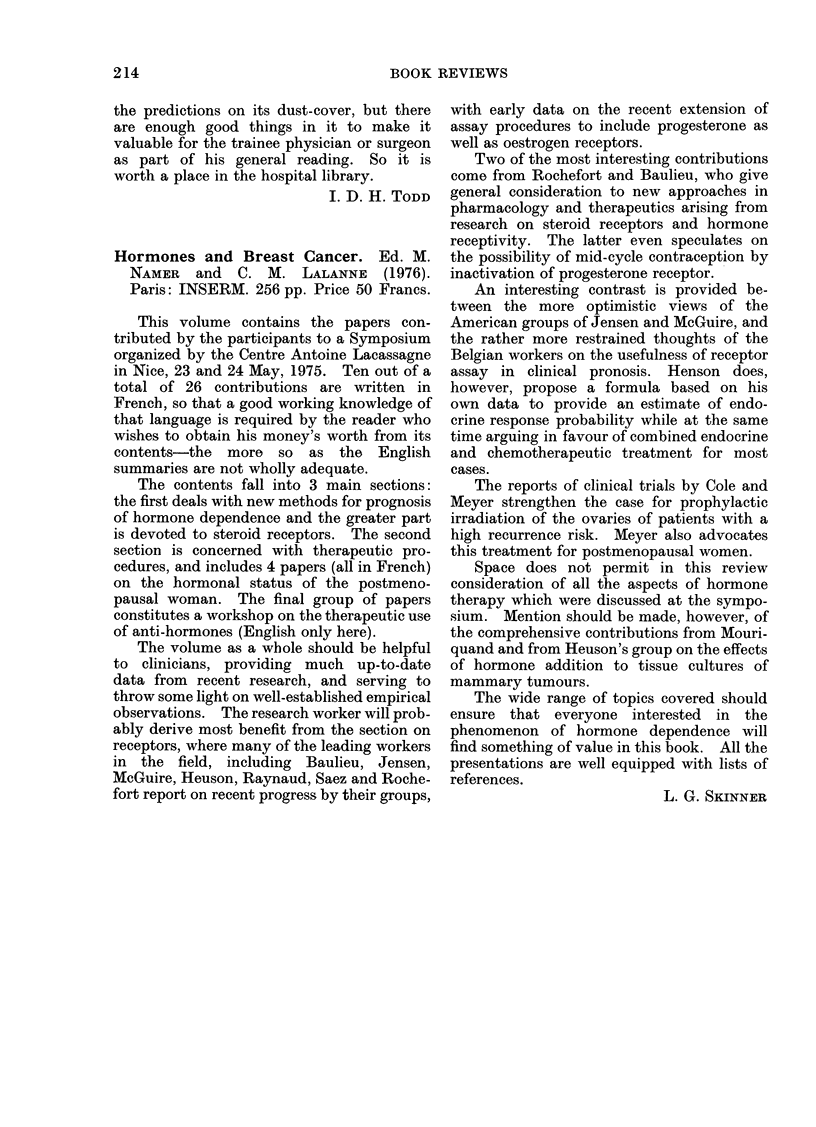# Radiotherapy in Modern Clinical Practice

**Published:** 1976-08

**Authors:** I. D. H. Todd


**Radiotherapy in Modern Clinical Practice.** Ed. H. F. HOPE-STONE (1976) Granada Publishing. x + 358 pp. Price £10.00 net.

Dr Hope-Stone has chosen a very difficult middle path for this book. It is relatively easy, although time-consuming, to edit an exhaustive text, more difficult to produce brief but balanced notes designed to help candidates for a specific examination, but most difficult of all to bring a very specialized subject to the more general reader who requires simplification and clarity without sacrificing too much of the contentious material which is inevitably associated with recent advances. This book is intended to help general physicians, surgeons and hospital doctors to understand something of the principles and practice of radiotherapy and of its place in the management of malignant disease, whilst at the same time " highlighting the advances which have been made in the last few years ".

The general chapters on the technical aspects of radiotherapy (B. S. Mantell), on radiobiological studies and clinical problems in radiotherapy (G. Wiernik), on fast neutron therapy and the oxygen effect (Mary Catterall), and on computing in radiotherapy (M. Cohen), are among the most effective and should be of most value to the general reader. The remaining chapters take the diseases by system or region and, although dealing mainly with radiotherapy, other aspects of oncology such as cytotoxic chemotherapy are included. The presentation is competent but variable, Hope-Stone's own chapters on tumours of the nervous system and the reticuloses being particularly well balanced. In most chapters adequate references are quoted to direct further reading. The consultant physician or surgeon who already has a general concept of the potentialities of radiotherapy in the common tumours is unlikely to find this book of help with rarities or unusual complications, but it should help the trainee who wants more general guidance. The reviewer is only too well aware of the speed with which the written word can date. The burning topic in the field of breast malignancy is the place of adjuvant chemotherapy in the management of the early case but this is not mentioned in the chapter on carcinoma of the breast, presumably as this controversy has been widely aired only in the past 2 years, after the manuscript had been passed to the publisher.

The production is beautiful, as it should be at the price. One of the few errors is that although hypernephroma is treated in the text on p. 286 the index refers one to p. 289.

This book does not quite measure up to the predictions on its dust-cover, but there are enough good things in it to make it valuable for the trainee physician or surgeon as part of his general reading. So it is worth a place in the hospital library.

I. D. H. TODD